# Comprehensive machine learning assessment of zebrafish behaviour and biochemical markers in response to caffeine exposure

**DOI:** 10.1007/s10646-025-02873-0

**Published:** 2025-03-19

**Authors:** Cláudia Teixeira, Sara Rodrigues, João Amorim, Bárbara S. Diogo, Ivo Pinto, António Paulo Carvalho, Sara C. Antunes, Luís Oliva Teles

**Affiliations:** 1 Departamento de Biologia, Faculdade de Ciências, Universidade do Porto, Porto, Portugal; 2 CIIMAR/CIMAR LA, Centro Interdisciplinar de Investigação Marinha e Ambiental, Universidade do Porto, Matosinhos, Portugal; 3 ICBAS, Instituto de Ciências Biomédicas de Abel Salazar, Universidade do Porto, Porto, Portugal; 4 UMIB, Unidade Multidisciplinar de Investigação Biomédica - Instituto Ciências Abel Salazar da Universidade do Porto (ICBAS), Porto, Portugal

**Keywords:** *Danio rerio*, Locomotor behaviour, Biochemical markers, Mahalanobis distance, Ecotoxicology, Integrated assessment

## Abstract

Environmental exposure to caffeine (CAF) poses potential risks to aquatic ecosystems, affecting non-target species. This study investigated the chronic effects of environmentally relevant CAF concentrations, ranging from 0.16–50 µg/L, on zebrafish behaviour. A Kohonen-type artificial neural network classified zebrafish behaviour into nine behavioural classes based on a set of movement descriptors (mean meander, mean velocity, instantaneous velocity, distance to centre point, mean angular velocity and instantaneous acceleration), while a comprehensive analysis integrated behavioural classes previously defined and biochemical markers of oxidative stress, lipid peroxidation, reserve energy content, energetic pathways, and neurotoxicity. The discriminant analysis demonstrated that behaviour descriptors and biomarkers individually explained 38% and 67% of data variation, respectively, while the combination resulted in 19 models with 100% correct diagnosis. One of the models (Model A) seemed to suit the best dose-response relationship, incorporating key biomarkers including superoxide dismutase, catalase, glutathione peroxidase activities, and behavioural characteristics such as movement distance and velocity. This suggested methodology offers a different approach to evaluating CAF’s ecological impact, highlighting behavioural analysis as a valuable complement to traditional ecotoxicological assessments. This study provides a novel framework for understanding organism-level responses to environmental stressors (e.g., several anthropogenic compounds), utilising Mahalanobis distance as an integrative response index. This approach shows promise for broader application in assessing the impact of various aquatic contaminants on aquatic organisms (from bacteria to fish), potentially extending to pharmaceuticals, pesticides, and industrial pollutants.

## Introduction

The increase of anthropogenic compounds in natural ecosystems can impact non-target species, compromising ecosystem functions and services. Caffeine (1,3,7-Trimethylxanthine - CAF) is the most consumed psychoactive compound in the world, with approximately 80% of the population consuming caffeinated products daily (Rosa et al. 2018; Burbano-L and Porfiri, 2020; Li et al. 2020). In humans, low doses of CAF can positively impact cognition, memory, and learning, while high doses induce mental impairment, hyperactivity, and anxiety responses (File et al. 1982; Fredholm et al. 1999; Angelucci et al, 2002; Smith et al. 2012). Caffeine stimulates the central nervous system (CNS), increasing alertness and decreasing fatigue in a dose-dependent manner (Einöther and Giesbrecht, 2013; Marin et al. 2011).

Caffeine is a suitable indicator for anthropogenic contamination of aquatic environments (Buerge et al. 2003; Ferreira et al. 2005). Due to its high usage, the introduction into the environment occurs through several routes, from excretion to poor disposal of products from various sources (Li et al. 2020). Its increasing consumption has resulted in higher detection levels in aquatic ecosystems, raising concerns about potential ecological impacts (Diogo et al. 2023). It has been reported that exposure to CAF can cause alterations to baseline behaviour in several fish species, leading to increased swimming activity, changes in feeding behaviour, and higher mortality rates (Christensen, 1981; Ulloa and Verreth, 2002; Steele et al. 2018; Vieira et al. 2018; Zhou et al. 2019). Zhou et al. (2019) reported higher angular velocity on zebrafish larvae (5 days post-fertilization, dpf) in locomotor activity at concentrations of 1, 10 and 100 µg/L, mainly in the dark regime. Ruiz-Oliveira et al. (2019) registered higher average speed and distance travelled in zebrafish adults exposed to CAF at 10 and 50 mg/L. Contrarily, Ladu et al. (2015) reported a decrease in velocity with increasing levels of CAF (5, 25 and 50 mg/L) in zebrafish. Similarly, Steele et al. (2018) reported that CAF exposure reduced the total distance travelled in zebrafish larvae, being most sensitive at a concentration of 0.039 mg/L. Analysis of the behaviours described in previous studies shows that CAF can act as an ecotoxic agent, affecting the nervous system and locomotor activity patterns of zebrafish.

Behaviour is the action or reaction to intrinsic or extrinsic circumstances to the animal (Scott and Solman, 2004; Hellou, 2011). Locomotor activity, in particular, is highly sensitive to environmental fluctuations, like temperature and food availability, making it a valuable indicator of an organism’s well-being (Lüersen et al. 2014). With technological advancements, video tracking systems have become powerful tools for studying behavioural responses to external changes, like contaminants (Guimarães et al. 2024), offering a cost-effective and efficient means of data collection. These systems have numerous advantages, as small recordings are sufficient, inexpensive, and easy to use (Xia et al. 2018), and have been applied to several species from bacteria to fish to assess behaviour alterations (Teles et al. 2015; Fernandes et al. 2016; Amorim et al. 2017; Xia et al. 2018). While CAF is widespread in aquatic environments, its long-term effects on non-target organisms’ behaviour remains poorly understood. An integrative approach combining behaviour analysis and biochemical markers can provide insights into CAF’s ecotoxicity. A multi-marker approach, involving different metabolic pathways and physiological functions, such as biomarkers of oxidative stress, lipid peroxidation, reserve energy content, energetic pathways, and neurotoxicity (Diogo et al. 2023), provides insights into both exposure and adverse effects of anthropogenic chemicals on organisms. Numerous studies have validated the sensitivity and reliability of biomarkers as effective tools for ecotoxicology (Abreu et al. 2018; Diogo et al. 2023). For example, assessing neurotoxicity is crucial in ecotoxicology, showcasing how contaminants can impact the nervous system of vertebrates, and acetylcholinesterase (AChE) is commonly used for this assessment. Disruption in AChE activity can lead to altered neural function, and impair motor coordination and overall fitness (Johann et al. 2021). Therefore, a combination of these approaches (behaviour *vs* biomarkers) could prove valuable with the added advantage that behaviour analysis is faster and less resource-intensive compared to biochemical markers determinations. Combining traditional biochemical markers analysis with behavioural assessment can enable predictive models for toxicology, and similar applications in other biological fields (Jones, 2008). Technological advances have allowed the development of model approaches, which can help decrease laboratory costs and waste while providing a more holistic approach (Ring et al. 2021; Pereira et al. 2024). Despite their potential, the development and evaluation of such models in ecotoxicology remain underexplored (Ring et al. 2021).

This work aimed to assess the chronic effects of environmentally relevant CAF concentrations on the locomotor behaviour of *Danio rerio* (zebrafish), by using a video-tracking system. Zebrafish are very active organisms, moving continuously during daylight, which is desirable for behavioural assays (Grillitsch et al. 1999; Teles et al. 2015). Zebrafish possess a well-characterized repertoire of social and defensive behaviours, helping in the characterization of their behavioural response to various stimuli (Gerlai, 2003). Their complex behavioural patterns can be modulated by psychoactive contaminants, like CAF (Maximino et al. 2011). Thus, this study also aimed to explore how biochemical markers (as reported in Diogo et al. 2023) enhance the interpretation of behavioural data and vice versa. Combining these approaches can bridge the gap between specific, sensitive biochemical markers and broader, ecologically relevant behavioural indicators. Thus, creating a predictive model involves selecting the most sensitive endpoints while minimizing the number of regressors.

## Materials & methods

### Chemicals and test solutions

Caffeine (molecular weight 194.19 g/mol) was acquired from Sigma Aldrich (CAS: 58-08-2; Sigma Aldrich; purity 99%). A stock solution (1000 μg/L) was prepared by diluting CAF in dechlorinated tap water. Eight concentrations were chosen to conduct this study, 0.00 (used as negative control, CTL), 0.16, 0.42, 1.09, 2.84, 7.40, 19.23, and 50.0 μg/L. These concentrations were selected given the environmental concentrations already reported for European surface waters (Zhou et al. 2019; Cerveny et al. 2022).

### Test organisms: zebrafish – origin, age, size, maintenance

Zebrafish (*Danio rerio*) is a freshwater cyprinid and standard model species used in many scientific areas, including ecotoxicology and behaviour (Rosa et al. 2018). Zebrafish used in this assay were obtained from a laboratory broodstock and reared under standard laboratory conditions in a zebrafish facility at CIIMAR - Interdisciplinary Centre of Marine and Environmental Research (Matosinhos, Portugal). Five-month old zebrafish with a length to the fork of 2.0 ± 0.05 cm and an initial weight of 0.15 ± 0.01 g were used. Fish were kept in 60 L tanks with aeration and dechlorinated tap water, with controlled conditions of photoperiod (16h^L^:8h^D^) and temperature of 27.0 ± 2.0 °C for three weeks (acclimatization period). Water quality parameters such as temperature, pH, dissolved oxygen, ammonium, and nitrite levels were monitored weekly. Fish were fed *ad libitum* with a commercial fish food (Tetra Goldfish) every 48 h. Trained researchers (following FELASA category C recommendations) directed the experiment, and all procedures were conducted according to the recommendations of the European Union Directive (2010/63/EU) while operating under the Portuguese Law (DL 113/2013) on the protection of animals for scientific purposes. The experimental protocol was approved by the Animal Welfare and Ethics Body committee of the Interdisciplinary Centre of Marine and Environmental Research (ORBEA-CIIMAR).

### Chronic ecotoxicity assay

The chronic assay was carried out according to OECD test guideline 215 (OECD 2000). This assay was performed under laboratory-controlled conditions similar to those adopted during the acclimatation period. Zebrafish were exposed for 28 days to eight concentrations of CAF (0.00, 0.16, 0.42, 1.09, 2.84, 7.40, 19.23 and 50.0 μg/L). Zebrafish were divided by twenty-four 4-L glass aquaria (randomly distributed in the exposure room), with three replicates per treatment (3 aquaria/replicate per treatment, each one with five fish) (for details *see* Diogo et al. 2023). The medium was 80% renewed every 48 h. Fish were kept in the same conditions as quarantine.

Physical and chemical water parameters (pH, temperature, and dissolved oxygen) were measured using a multiparametric probe (Multi 3630 IDS SET F), and for ammonium and nitrites determinations, water aliquots were collected, before medium renewal, and a bench photometer (Spectroquant Multy Colimeter) was used for the nutrient quantifications. These parameters were quantified twice per week for validation assay purposes (more details and results, *see* Diogo et al. 2023). The concentrations previously mentioned are nominal, but analytical concentrations were determined using high-pressure liquid chromatography (UPLC-MS/MS) (*see* detailed methodology in Diogo et al. 2023). Analytical determinations at the beginning of the assay (0 h) were below the quantification limit (BLOQ) in the CTL treatment while the remaining CAF concentrations were: 1.67 ± 0.18, 2.03 ± 0.07, 3.47 ± 0.29, 9.03 ± 0.50, 22.67 ± 2.33, and 60.67 ± 2.73 µg/L. After 48 h of exposure and before the medium was renewed, the analytical concentrations were BLOQ (CTL), 0.78 ± 0.07, BLOQ, 0.64 ± 0.24, 2.47 ± 0.82, 8.27 ± 2.03, and 38.67 ± 0.67 µg/L.

### Behaviour evaluation: video-tracking

#### Experimental plan

Swimming behaviour was evaluated using a custom-made video-tracking system for recording, followed by analysis with an adapted algorithm for determining the centre of fish mass. The video-recording system used was similar to the one described by Teles et al. (2015). Recordings were performed using four Flow Electronics 540 L IR with CCD 1/3″ Sony sensor, resolution 795 × 596 PAL (model: CACO0008) cameras connected to Camtronics DVR 38 AHD PLUS capture device. Cameras were placed in an isolated temperature- and light-controlled recording chamber. Each camera filmed a total of six circular arenas. Three fish from each experimental condition were randomly selected and used in the behavioural assessment. Each fish was placed, individually, in a circular arena with 150 mL of system water (i.e. CAF concentration). Only horizontal movement was recorded, as the depth of the arenas was reduced to a minimum value (5 cm). All fish were always randomly distributed through the four cameras (24 arenas) to avoid potentially biased phenomena such as poor peripheral tracking. Fish were filmed for 45 min, comprising 15 min of acclimatization to the arenas and 30 min of recording (Amorim et al. 2017).

#### Image processing

Video processing was performed on an Intel® -Core™ i7-2600K CPU (@3.40 GHz, 16.00 GB RAM) computer with Windows 10Pro. Recorded videos were processed in Matlab software (2017a) using the in-house adapted algorithm (Román et al. 2018; Guimarães et al. 2024). The algorithm works by autonomously detecting the circular arenas, tracking and analysing the animals’ trajectories, and validating and extracting behavioural parameters. The algorithm works through three distinct phases, each responsible for different parts of the video processing and analysis (described in Guimarães et al. 2024). From the Matlab (2017a) algorithm, six movement descriptors were calculated: mean meander (degrees/mm), mean velocity (mm/s), instantaneous velocity (mm/s), distance to the centre point (mm), mean angular velocity (degrees/s), and instantaneous acceleration (mm/s^2^).

### Statistical analyses

**Fig. 1:**
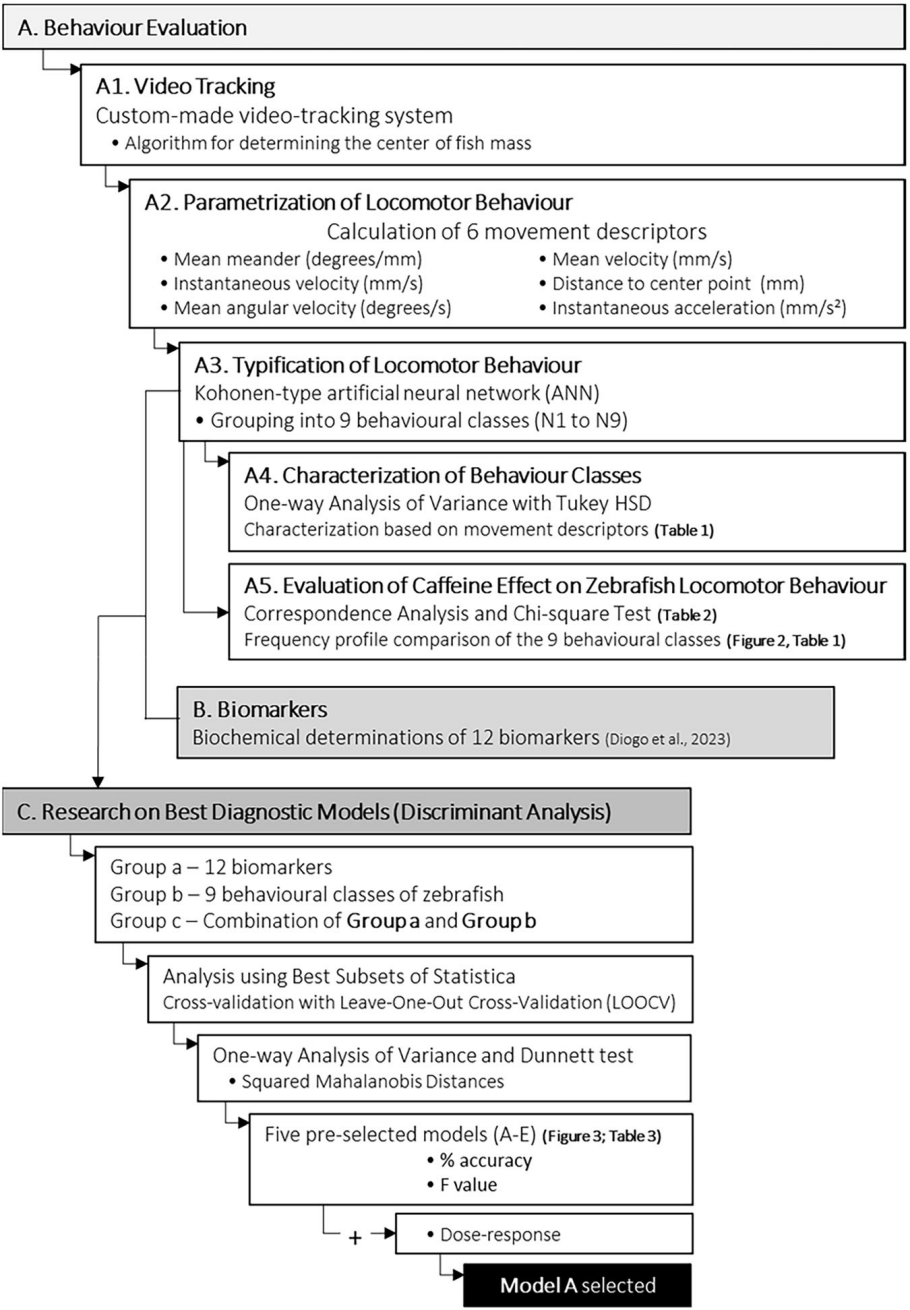
Sequential flowchart illustrating the statistical analysis of zebrafish behaviour and biomarkers (Diogo et al. 2023) results after chronic caffeine exposure

A Cluster Analysis was used to reduce behavioural variables from those calculated by the algorithm and create behavioural classes (Fig. 1). The variables employed for the behavioural analysis (movement descriptors) encompassed mean meander (degrees/mm), mean velocity (mm/s), instantaneous velocity (mm/s), distance to the centre point (mm), mean angular velocity (degrees/s) and instantaneous acceleration (mm/s^2^) (Table 1). Nine behavioural classes (N1 to N9) were defined using a Kohonen-type artificial neural network (ANN) that grouped each frame of the fish into nine different behavioural classes (characterized by different behavioural attributes) (Fig. 1; Table 1). The ANN groups data by similarity, reducing complexity and maintaining analysis resolution, and this ANN works without supervision (Qiao and Han, 2019; Fernandes et al. 2016; Amorim et al. 2018). One-way Analysis of Variance with the Tukey HSD was used to characterise the behavioural classes (Fig. 1). The movement descriptors used homogenous groups (that reflect similar behavioural effects) to describe the behavioural classes (Table 1).

**Table 1:** Description of the behavioural classes

Behavioural classes	Behavioural attributes	Movement Descriptors					
		Mean meander (degrees/mm) F_[8, 24,530]_ = 7094.5; *p* < 0.001	Mean velocity (mm/s) F_[8, 24,530]_ = 15,271; *p* < 0.001	Instantaneous velocity (mm/s) F_[8, 24,530]_ = 15,868; *p* < 0.001	Distance to centre point (mm) F_[8, 24,530]_ = 2451; *p* < 0.001	Mean angular velocity (degrees/s) F_[8, 24,530]_ = 7042.3; *p* < 0.001	Instantaneous acceleration (mm/s^2^) F_[8, 24,530]_ = 1876.5; *p* < 0.001
N1	Lowest Distance to the centre.	12.9^c^	13.3^d^	13.0^c^	30.5^a^	43.7^b^	−0.4^c^
N2	Highest Distance to the centre. Lowest angular velocity.	43.7^d^	3.5^b^	2.6^a^	40.6^d.e^	26.2^a^	−0.6^c^
N3	Average values of movement descriptors^1^	3.7^a.b^	29.3^f^	35.1^g^	40.1^c^	56.6^d^	5.6^d^
N4	Highest Distance to the centre.	5.5^b^	34.0^g^	33.9^f^	40.6^d^	95.4^f^	1.5^c^
N5	Highest Meander. Lowest mean and instant velocity.	186.0^f^	0.6^a^	0.8^a^	39.7^b.c^	48.4^c^	0.3^c^
N6	Lowest Instant acceleration.	3.9^a.b^	23.5^e^	15.5^d^	39.9^c^	49.9^c^	−7.8^b^
N7	Highest Distance to the centre, mean, instant, and angular velocity. Lowest Meander.	3.2^a^	50.7^i^	59.1^h^	41.0^e^	96.6^g^	9.8^e^
N8	Highest Distance to the centre, angular velocity. Lowest instantaneous acceleration.	4.4^a.b^	44.2^h^	16.6^e^	40.7^d.e^	100.1^g^	−36.0^a^
N9	Average values of movement descriptors^2^.	55.4^e^	5.9^c^	4.4^b^	38.9^b^	77.0^e^	−0.5^c^

Mean values of the movement descriptors in the nine behaviour classes defined by *ANN* (artificial neural network) and respective homogeneous groups. Different letters (in superscript) stand to discriminate the differences between behavioural classes (Tukey Unequal N HSD post-hoc test, significance was accepted when *p* < 0.05)

^1^N3 is characterized by lower average values of meander, higher average values of mean velocity and instantaneous velocity, and instantaneous acceleration, the highest contributors to the behavioural class. ^2^N9 is characterized by higher average meander, lower mean and instantaneous velocity as well as instantaneous acceleration, which are the highest contributors to this behavioural class

A Correspondence Analysis and a Chi-square test of independence (Table 2) were subsequently carried out to investigate possible differences between experimental conditions (Fig. 1). A residual analysis was then carried out to explore specific differences among treatments; the statistical significance of each residual was determined by comparing the respective Chi-square contribution (i.e., partial χ2 value) against the critical distribution value determined with the Bonferroni correction for the total number of cells (Table 2). This evaluation compared the frequency profile of fish across the nine locomotor behavioural classes with their CAF exposure levels, including the control group. The frequency profile was calculated by determining the behaviour class of the fish at each frame using the ANN.

**Table 2:**
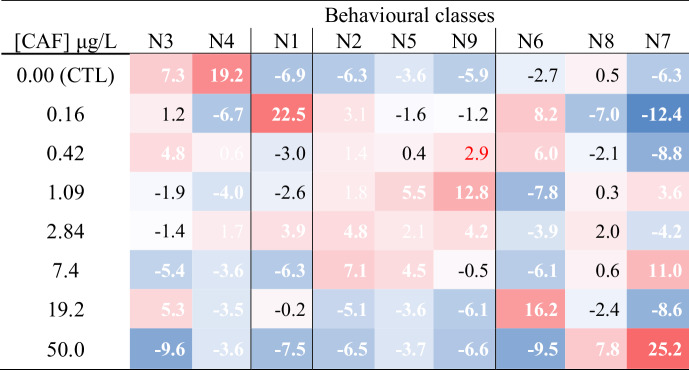
Chi-square standardised residual values observed in the nine behaviour classes (N1 to N9, see Table 1) defined by the Kohonen-type ANN given the experimental conditions

Significant differences (*p* < 0.05) are highlighted in bold, following the Bonferroni correction for expected values. Colour intensity represents positive deviations (red) and negative deviations (blue). Bold white values appear to be characteristic of the behavioural class; in bold significant following the Bonferroni correction regarding expected value. Classes were reorganized for a better depiction of the effect of caffeine on the behaviour

To explore the relationship between CAF concentrations (dependent variable, Y) and the combination of biochemical markers and behaviour (independent variables, X), three discriminant analyses (DA) were conducted (Fig. 1). The first conducted to the 12 assessed biochemical markers (results at Diogo et al. 2023) (group a), the other containing the nine behavioural classes obtained from the ANN (group b), and lastly, the third one combining both types of biological responses (12 biomarkers and 9 behavioural classes) (group c) (Fig. 1). The best diagnostic model was identified using Discriminant Analysis via the Best Subsets option in Statistica v13 (TIBCO Statistica, StatSoft GmbH, Germany). The criterion for selecting the best predictor combination was the classification error rate from cross-validation (Leave-One-Out Cross-Validation, LOOCV). For each group with only behavioural classes and biomarkers, an automatic search of all possible predictor combinations was feasible, however, the same could not be done for the 21 regressors in the last DA. Therefore, searches with a fixed number of predictors (ranging from 1–21) were conducted. A One-way ANOVA followed by a Dunnett test was performed on the exposure levels using Squared Mahalanobis Distances (Fig. 1) of each validated point relative to the control group’s centroid.

All statistical analyses were performed using Statistica v13 (TIBCO Statistica, StatSoft GmbH, Germany) for Windows (Fig. 1), and all significant differences were considered when *p* < 0.05.

## Results and discussion

No mortality was observed in the control group during CAF exposure, complying with the OECD 2000 guidelines. Water quality parameters were measured during the exposure period, and ammonium and nitrites were maintained at residual levels (Diogo et al. 2023). Regarding the literature, the highest CAF concentrations encountered in the environment were 1.1 mg/L in surface waters and 3.6 mg/L in wastewater (Spongberg et al. 2011; Tran et al. 2014, respectively). However, the highest values registered in Europe do not surpass 39.81 µg/L in surface waters (Zhou et al. 2019). So, the tested concentrations are below the European values, except for the highest concentration at 50 µg/L, showing the ecological relevance of the present study.

**Fig. 2:**
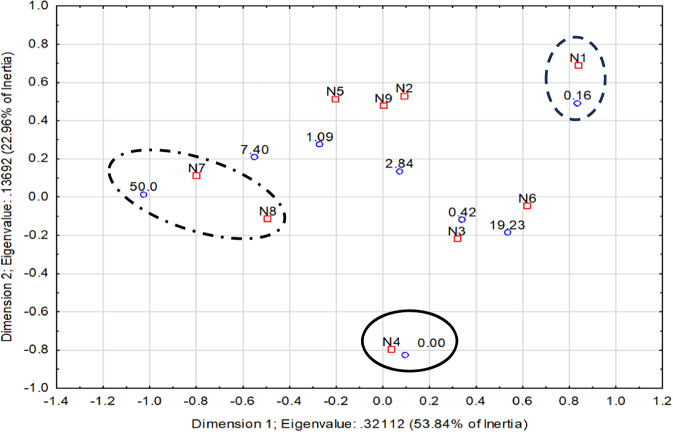
Summary of statistics 2D plot, regarding CAF concentrations (0.00–50.0 μg CAF/L - blue triangles) and behavioural classes (N1 to N9 – red squares) results:  highest distance to the centre;  highest mean velocity and mean angular velocity;  low distance to the centre

The ANN identified nine behavioural classes (N1 to N9) (Fig. 2; Table 1) and the average values for each movement descriptor are described in Table 1. The frequency profile of the nine behavioural classes resulted in an inertia value of 59.6% with the experimental conditions tested (eight CAF concentrations after 28 days of exposure). A highly significant chi-square test (χ2 = 3820.0; d.f. = 56; *p* < 0.001) confirmed that all behavioural classes were affected by CAF exposure, with observed frequencies differing from expected values (Table 2). Specifically, the locomotor behaviour class N4 was associated to the control group; class N1 to the lowest concentration (0.16 µg/L); classes N2, N5, and N9 to concentrations between 1.09 and 7.40 µg/L; class N6 at the second highest concentration (19.2 µg/L). Classes N7 and N8 were correlated with the highest concentration (50.0 µg/L) (Table 2 and Fig. 2). The highest deviation (25.2) in behaviour frequency is observed in class N7, in fish exposed to the highest concentration of CAF (Table 2). This behaviour is characterized by having the highest average values in all movement descriptor parameters of the fish, except for the mean meander, which shows the lowest average value (Table 1). The behavioural classes N8 and N6, positively associated with the highest and second highest (50 and 19.23 µg/L) concentrations of CAF respectively are mainly characterized by high negative instantaneous accelerations. The behaviour class N4, positively associated with the control (0 µg/L), is mainly characterised by having high average instantaneous acceleration, but not as high as those observed in behaviour class N7 (Table 1). The remaining behavioural classes are mainly characterized by having the lowest average value of distance to the centre (N1 - positively associated with the 0.16 µg/L concentration); and the highest average values of mean meander (N2, N5, and N9 - positively associated with concentrations between 0.16 and 7.40 µg/L, Table 2).

Present results corroborate previously reported works, where higher swimming activity has been described for other toxicants closer to the no observed adverse effect concentration (NOAEC) values (Magalhães et al. 2007; Amorim et al. 2017). Nonetheless, behavioural classes N3, N4, N7 and N8 have the lowest values of meander (Table 1), which could be a direct consequence of speed. However, only N7 and N8 have the highest and second-highest mean velocity, respectively. These behavioural classes are highly correlated with the highest tested concentration (50 µg/L). As CAF is a stimulant of the CNS, it can increase activity, which can be translated into an increase in velocity and swimming speed, as it works in a dose-response relationship (Marin et al. 2011; Einöther and Giesbrecht, 2013; Steele et al. 2018; Ruiz-Oliveira et al. 2019; Zhou et al. 2019). Due to inertia, meander, which measures the curvature of an object’s movement, tends to decrease as the swimming speed of fish increases. Contrarily, behavioural classes N2, N5 and N9 showcase higher mean meander values, in contrast to mean velocity. Similar studies have already observed this in zebrafish exposed to nanoparticles and anthropic toxicants (Li et al. 2017; Amorim et al. 2017, 2018; Capriello et al. 2019; Krylov et al. 2020).

Zebrafish have already been exposed to CAF, to understand alterations in the baseline behaviour, namely in object discrimination (Santos et al. 2016), alertness and concentration, and decrease in fatigue (McLellan et al. 2016). However, higher concentrations have led to hinder these effects, 14 mg/L in jewel fish, >40mg/day in humans, and 4 mg/kg in humans (Burgess, 1982; Rogers et al. 2013; McLellan et al. 2016, respectively) and could lead to adverse outcomes, such as tachycardia, increased ventilation, restlessness, insomnia, and anxiety (Benowitz, 2008; Butt and Sultan, 2011; Franco et al. 2013; Ardais et al. 2014; Rosa et al. 2018). Moreover, other studies have indicated that high doses of CAF (>20 mg/L) can induce adverse effects on locomotor activity of zebrafish (Richendrfer et al. 2012; Ladu et al. 2015), exacerbate anxiety-like behaviour, and potentially decrease locomotion (El Yacoubi et al. 2000; Hughes and Hancock, 2016; Santos et al. 2017; de Carvalho et al. 2019). Regarding these results, further studies should be conducted to understand the underlying mechanisms present in fish, especially in neurotransmission, since CAF can bind to adenosine receptors and block specific neurotransmission pathways in other species (Daly et al. 1994). However, the exact effects of CAF on fish neurotransmission remain unclear and could differ due to species-specific variations in receptor distribution and function. Future research should focus on receptor-binding studies to identify how CAF interacts with adenosine receptors in fish, and investigate the molecular pathways involved in its effects. This could involve both in vitro and in vivo approaches to assess receptor affinity, signalling cascades, and the physiological outcomes of CAF exposure. Indeed, CAF ‘s effect on behavior depends on the blockage of the A1 and A2 adenosine receptors (Santos et al. 2023), and more studies should also be conducted to recognize how these CNS stimulants can affect the behaviour of non-target aquatic species.

To better understand the underlying mechanisms, a Discriminant Analysis (DA) (Fig. 1) was used to study the relationship between fish exposure to CAF and locomotor behaviour, complemented with the biochemical markers results (Diogo et al. 2023). A set of 12 biomarkers were chosen for assessed the activity of antioxidant defences such as superoxide dismutase (SOD), catalase (CAT), glutathione peroxidase (GPx), Glutathione Reductase (GRed); glutathione (GSH) content; glutathione-S-transferase (GSTs) activity; lipid peroxidation through thiobarbituric acid reactive substances (TBARS) levels; glycogen (GLY), lipids (LIP) and total protein (PROT) contents; as well as lactate dehydrogenase (LDH) and AChE activities (Diogo et al. 2023). The joint evaluation of these biomarkers allows a better understanding of the effects of CAF on the organism and the underlying mechanisms. For DA, the 12 biomarkers (Diogo et al. 2023) and the 9 behavioural classes (Table 1) were used to find the linear combination that best explained the relationship between these variables and CAF exposure.

Through DA, for the biochemical biomarkers (group a) and the behavioural classes (group b), a comprehensive search of all possible combinations among their respective predictors was automatically conducted using the best subset option. The criteria used to select the best combination was the cross-validated data classification error rate, through the leave-one-out cross-validation (LOOCV). In the DA with the twelve biochemical biomarkers (group a), models achieving up to 67% correct diagnoses were found in 8 models using 5–9 biomarkers as predictors. The DA of the nine behavioural classes (group b) achieved 38% correct diagnoses in one model containing three behavioural classes as predictors. Due to the vast number of potential combinations, the same was not feasible when combining both biological responses (group c). To partially circumvent this limitation, within this group of regressors, 21 searches were conducted with a fixed number of regressors, ranging from 1 to the total number of regressors in this group (21). The best response was obtained when joining both, in which 19 models had 100% correct diagnosis, with 12–15 regressors. The number of regressors in these models was relatively high, which may increase suspicion of overfitting of the model. However, this doesn’t seem to be the case. Firstly, overfitting is usually related to the training dataset, not the validation dataset. The inflection point in the model’s performance occurs when the number of regressors exceeds 15. This suggests that the model begins to overfit only when this threshold is reached.

**Fig. 3:**
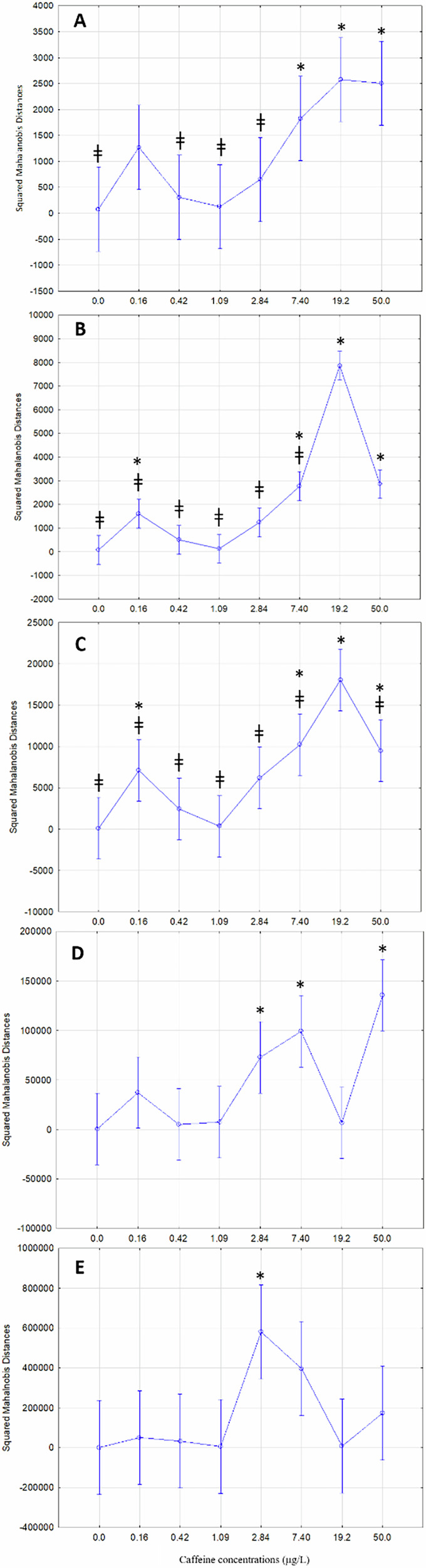
Graphical representation of the 5 significant models (**A**–**E** – data included behavioural responses and biomarkers results, *see* Table 3) of the Squared Mahalanobis Distances plotted with the caffeine treatments. * represents significant differences between control, and ǂ stands for differences between 19.2 μg CAF/L (Dunnett’s test *p* < 0.05)

To select the best model among the 19 models with 100% correct diagnosis previously selected, the Mahalanobis squared distance was estimated between the experimental conditions (test groups) and their respective controls for each model. The Mahalanobis distance (Mahalanobis, 1930) is a measure that considers the correlation between variables and is used in the DA to calculate the probability of an unknown element belonging to a specific group or class (Xiang et al. 2008). An analysis of variance (ANOVA) with Dunnett’s post-hoc test was conducted to analyse the relationship between Mahalanobis distances and the conditions to which the fish were exposed in the 19 previously selected models. Among these, only 5 models (A, B, C, D, and E) showed statistically significant effects (Table 3). Given the premise that CAF exhibits a monotonic dose-response relationship, common to most non-carcinogenic substances (Migita et al. 2009; Reggiani, 2021), models A, B, and C are the models that best adhere to this premise (Fig. 3, Table 3). In the three models, the first exposure concentration (0.16 µg/L) is higher than the control and the following three concentrations (0.42–2.82 µg/L), which could be explained through the not-uncommon phenomenon of hormesis. Hormesis is a phenomenon in which a harmful substance stimulates the organism when exposed to low concentrations (Sakai, 2006), referring to an adaptative response of biological systems to challenges (Calabrese and Mattson, 2017). The physiological implications of hormesis in zebrafish exposed to CAF include stimulating activity at low concentrations, and improving swimming or foraging, but leading to a potential neuromuscular inhibition and stress at higher doses. Ecologically, this may cause an increased risk of predation due to hyperactivity or population decline at high concentrations, affecting the ecological balance and food chain dynamics in contaminated waters. Among these three models, models B and C also seem to contradict the expectations of a dose-response relationship, despite having the highest Fisher’s F values (Table 3). This is because the Mahalanobis distances at the highest concentration show a sharp decrease, which should at least be non-significant relative to the previous concentration (Fig. 3). Contrary to expectations of a dose-response relationship, particularly with models B and C, where Mahalanobis distances decrease unexpectedly at higher concentrations, could indicate non-monotonic dose-response effects, for higher concentrations. This phenomenon, where the effect does not follow a simple increase or decrease with concentrations, may reflect complex biological interactions. In model A, the slight nonsignificant decrease in the Mahalanobis distance observed at the last test concentration suggests that the effect of CAF may have reached a plateau from the concentration of 7.40 µg/L (Fig. 3), as confirmed by the Dunnett test against the concentration of 19.2 µg/L, which has the highest Mahalanobis distance. For this reason, model A seems to be the model that best represents the dose-response relationship between fish exposure to CAF and fish locomotor behaviour. Table 3 summarises the best models achieved with the statistical analysis of the behavioural classes and biomarkers results measured in zebrafish, after chronic exposure to a range of CAF concentrations. In models B and C, the decline in Mahalanobis distances between 19.2 and 50.0 µg/L is much more significant, to the point where both concentrations have statistically significant mean distances by a Dunnett test applied against 19.2 µg/L, which is the concentration with the highest mean Mahalanobis distance. This statistically significant decrease in Mahalanobis distance goes against expectations, given that concentration 50.0 µg/L is the highest concentration, and these distances measure the distance of each test concentration to the control group.

**Table 3:** Summary of the description of the significant models regarding behavioural classes (*see* Table 1) and biomarkers results (Diogo et al. 2023)

Model	Behavioural Classes									Biomarkers											N° of effects	Accuracy (%)	F_[7, 16]_	*p*
A	N1	N2	N3			N6				SOD	CAT	GPx			TBARS	Gly	Lip		LDH	AChE	12	100	7.31	**<0.001**
B	N1	N2				N6		N8		SOD	CAT	GPx			TBARS	Gly	Lip		LDH	AChE	12	100	79.8	**<0.001**
C	N1	N2				N6				SOD	CAT	GPx	GSH	GSTs	TBARS	Gly	Lip		LDH	AChE	13	100	11.7	**<0.001**
D		N2	N3	N4	N5		N7		N9	SOD	CAT	GPx		GSTs	TBARS	Gly	Lip	Prot			14	100	9.10	**<0.001**
E	N1	N2	N3		N5		N7		N9	SOD	CAT	GPx		GSTs	TBARS	Gly	Lip	Prot			14	100	3.86	**0.012**

One-way ANOVA with the Dunnett’s post-hoc test was applied to the Squared Mahalanobis distances, significance was accepted when *p* < 0.05

Models A and B are both composed of the same set of eight biomarkers (SOD, CAT, GPx, TBARS, Gly, Lip, LDH, and AChE) (Table 3). Model C is composed of the same biochemical markers set but also GSTs and GSH (Table 3), and in total, it is formed by one more regressor than the remaining two models (12 for 13 number of effects). These models also comprise the behavioural classes N1, N2, N3, N6 for model A and N1, N2, N6, N8 for model B. Behavioural classes N1, N2, and N6 are also integrated model C (Table 3). Notably, behavioural classes N7, associated with the highest concentration (50 µg/L), and N4, associated with the control group (Fig. 2), didn’t integrate the final models. Especially when behaviour N7 was the class that most contributed to the Chi-square test (Table 2) both these conditions discriminate extremes of CAF concentrations, therefore the main contribution of the behaviour seems to discriminate the intermediate exposure conditions, which are more challenging.

The biomarkers that make up the final model appear to complement behaviour in assessing the health status of zebrafish. Evidence of relation between AChE activity and behaviour has already been reported in several species from springtails (Isotomidae family) to rats (Paul et al. 1998; Dam et al. 2000; Mesquita et al. 2011; Pereira et al. 2013; de Oliveira et al. 2023). Alterations of locomotor behaviour are often linked with AChE inhibition (Andrade et al. 2016). Haskell-Ramsay et al. (2018) identified CAF as a substance that inhibits enzymes responsible for the breakdown of neurotransmitters, namely AChE. The inhibition of AChE activity at the highest concentrations (19.2 and 50.0 µg/L; *see* Diogo et al. 2023) is characteristic of fish with more significant behavioural disturbances (N8 and N9 behavioural classes; Fig. 2; Table 1).

Oxidative stress may also be responsible for neurobehavioural disorders (Lee et al. 2020), and therefore SOD, CAT, and GPx activities evaluation may be helpful in the defence against oxidative stress (Santos et al. 2018; Audira et al. 2020). Oxidative stress scenarios can be related to damaged lipids since free radicals are not being combatted and neutralized effectively by antioxidant defences, which can provide information on the health status of organisms(Lesser, 2012). Diogo et al. 2023, showed that SOD activity tended to increase with CAF concentrations (except 19.2 µg/L), which can superimpose/potentiate SOD antioxidant capacity to combat superoxide radicals. The activity of SOD is crucial for protecting against oxidative stress, which can affect muscle function (free radicals like superoxide radicals can damage muscle cells), neurological health (e.g. neurons involved in motor control), energy metabolism (e.g. disruption of mitochondrial function), and overall stress response, all of which are critical for normal swimming behaviour in aquatic organisms (Santos et al. 2018; Audira et al. 2020; Diogo et al. 2023).

Lactate dehydrogenase is a biochemical marker of chemical and environmental stress, which can compromise behaviour due to the metabolic costs involved in detoxification (Andrade et al. 2016; Santos et al. 2018), representing an anaerobic pathway for energy production. The general finding is that as CAF concentrations increase, organisms may adjust energy reserve and metabolic pathways to combat oxidative stress (significant decrease in LDH activity > 0.42 μg/L; Diogo et al. 2023). These biomarkers can provide additional information on the health status of the fish, demonstrating that CAF could cause disruptions that can collectively or individually affect muscle function, energy metabolism, and neural control. Fish exposed to CAF manifest altered swimming behaviour, in environmentally relevant concentrations, reflecting the organism’s compromised physiological state and health. However, further research should focus on understanding the modes of action and secondary events linked to toxicant response, allowing for an environmental diagnosis and monitoring, as well as their implications for ecosystem health.

By reducing the number of responses (biomarkers and behavioural classes – Table 3), we want to create a more generalizable and robust predictive model. This approach lets us focus on the most relevant and informative parameters, avoiding potential noise and unnecessary complexity. A simpler model is also easier to implement in practical applications and requires fewer resources to maintain and update, especially concerning biomarkers. Overall, obtaining a predictive model with the least possible number of responses helps strike a balance between accuracy and simplicity, making it more valuable and applicable in real-world scenarios.

## Conclusions

Overall, our study reaffirms zebrafish as a model organism for analysing behaviour and ecotoxicological effects of anthropic compounds. Additionally, it was demonstrated that even lower CAF concentrations (environmentally relevant) can cause noticeable behavioural effects in this species. Moreover, it was shown that locomotor behaviour is a sensitive ecotoxicological tool and can slightly reduce the number of biomarkers commonly used in ecotoxicological assays. This brings advantages not only in efficiency but also in reducing the number of animals sacrificed for biomarker analysis, and evaluation for long-term exposure, as well as a decrease in costs and time to obtain results. Besides demonstrating high predictive capability, the regression models used in this study have the significant advantage of providing an integrated measure of the organism’s response to the conditions they are exposed to, using Mahalanobis distances. Future work should be carried out with other xenobiotics and other biological biomarker models to better understand the applicability of the Mahalanobis distance as a reliable index for ecotoxicological assays.

## Data Availability

Data will be made available upon request.
